# Deletion of RAGE Causes Hyperactivity and Increased Sensitivity to Auditory Stimuli in Mice

**DOI:** 10.1371/journal.pone.0008309

**Published:** 2009-12-15

**Authors:** Seiichi Sakatani, Kazuyuki Yamada, Chihiro Homma, Seiichi Munesue, Yasuhiko Yamamoto, Hiroshi Yamamoto, Hajime Hirase

**Affiliations:** 1 Hirase Research Unit, RIKEN Brain Science Institute, Wako, Saitama, Japan; 2 Research Resource Center, RIKEN Brain Science Institute, Wako, Saitama, Japan; 3 Department of Biochemistry and Molecular Vascular Biology, Kanazawa University Graduate School of Medical Science, Kanazawa, Japan; 4 Saitama University Brain Science Institute, Saitama, Japan; Case Western Reserve University, United States of America

## Abstract

The receptor for advanced glycation end-products (RAGE) is a multi-ligand receptor that belongs to the immunoglobulin superfamily of cell surface receptors. In diabetes and Alzheimer's disease, pathological progression is accelerated by activation of RAGE. However, how RAGE influences gross behavioral activity patterns in basal condition has not been addressed to date. In search for a functional role of RAGE in normal mice, a series of standard behavioral tests were performed on adult RAGE knockout (KO) mice. We observed a solid increase of home cage activity in RAGE KO. In addition, auditory startle response assessment resulted in a higher sensitivity to auditory signal and increased prepulse inhibition in KO mice. There were no significant differences between KO and wild types in behavioral tests for spatial memory and anxiety, as tested by Morris water maze, classical fear conditioning, and elevated plus maze. Our results raise a possibility that systemic therapeutic treatments to occlude RAGE activation may have adverse effects on general activity levels or sensitivity to auditory stimuli.

## Introduction

The receptor for advanced glycation end-products (RAGE) is a multi-ligand receptor that belongs to the immunoglobulin superfamily of cell surface receptors [1], [2]. A full-length RAGE has one transmembrane domain and the extracellular region contains one V-type and two C-type immunoglobulin (ligand binding) domains [1]. In situ hybridization and RT-PCR studies suggest a widespread existence of RAGE in body organs with the highest expression level in the lung [3]. In addition to the full length RAGE, various splice variants have been identified including the endogenous secretory form of RAGE (esRAGE) which may act as a decoy receptor in extracellular space [4], [5].

Ligands of RAGE include high mobility group box 1 (HMGB1, also known as amphoterin) [6], amyloid β-peptide (Aβ) [7], and S100B [8]. These ligands are known to be upregulated in neuropathological conditions. For instance, accumulation of Aβ occurs from an onset of Alzheimer's disease [9]. HMGB1 and S100B levels are increased in neuroinflammatry conditions such as in epilepsy and ischemia [10], [11]. Interestingly, S100B knockout mice have been reported to enhance spatial memory and context dependent fear memory [12]. Recently, S100B-RAGE interaction has been implicated in the brain *in vivo* in a condition that mimics epileptic seizures by kainic acid administration [13].

RAGE KO mice have been generated by a multiple number of laboratories [14], [15]. Although RAGE KO mice have been utilized in biochemical and physiological experiments to address the roles of RAGE in progression of various pathological conditions, consequences of lacking RAGE in normal condition have hardly been addressed. In this study, we performed a series of standard behavioral tests to identify the phenotype of RAGE KO mice.

## Results

**Figure 1:**
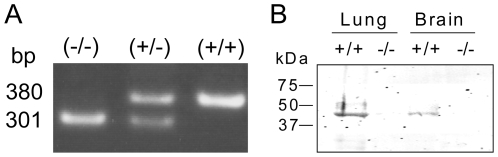
RAGE deletion in RAGE KO mice was confirmed by both DNA and protein levels. (A) PCR for ear samples shows RAGE(−/−) mice have a single band at 301 bp, RAGE(+/+) mice have a single band at 380 bp, and RAGE(+/−) mice have both bands, as described in Myint et al. [14]. (B) Western blotting analysis shows that RAGE is present in both the lung and brain in a RAGE(+/+) mouse.

Prior to the behavioral experiments, genotyping was performed by PCR (Fig. 1 A) and the body weight was measured for each mouse. Two sets of behavioral experiments with different sets of mice were performed to assure the results. The first set of animals (Set 1) consisted of a WT population (n = 10) that weighed 27.42±3.66 g and a RAGE KO population (n = 10) that weighed 26.88±2.73 g. The second set of mice (Set 2) consisted of a WT population (n = 10) that weighed 23.61±1.16 g and a RAGE KO population (n = 10) that weighed 22.27±1.33 g. There was no significant difference in the mean body weight between WT and KO (t-test, p = 0.713) in Set 1, however, the mean body weight was significantly different in Set 2, (t-test, p<0.05), although the difference was small. There were no mice with obvious abnormal appearance. In some mice, genotypes were reconfirmed at the protein level by Western blotting (Fig. 1B).

**Figure 2:**
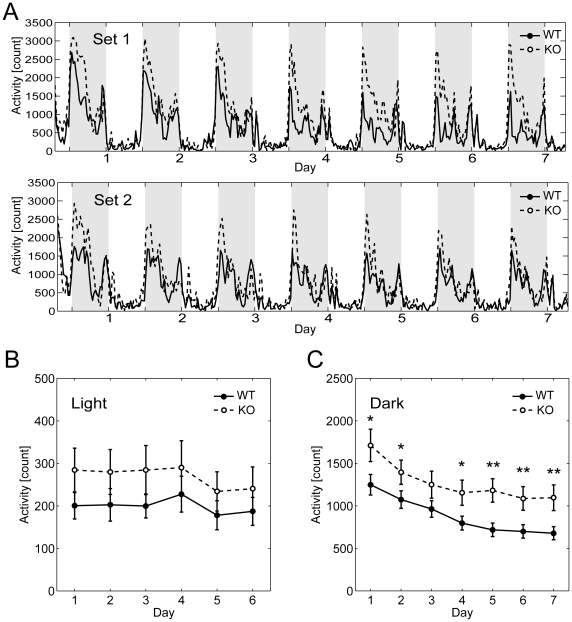
Enhanced home cage activity in RAGE KO mice. (A) Average home cage activity records of WT (solid line) and RAGE KO (dashed line) mice are shown for two independent sets of experiments (see main text for more details). The light and dark phases are indicated by white and grey backgrounds, respectively. (B) Group comparison of home cage activity in the light phase. The activity in the light phase is similar and remained constantly low. (C) Group comparison of home cage activity in the dark phase. The activity of KO mice in dark phase is higher than that of WT mice. Note that both WT and KO showed a gradual decrease in activity in the dark during the course of the seven days. For B and C, Set 1 and Set 2 are combined. Data are mean±S.E.M. * p<0.05, ** p<0.01.

The mice were assessed for home cage activity. As the room illumination is controlled at 12/12 hour light/dark cycle, the animals' activity was modulated accordingly with more activity during the dark phase (Fig. 2A) During the seven days of continuous monitoring, KO displayed more activity than WT (two-way ANOVA with repeated measurements for genotype, F(1,18) = 6.426, p<0.05 for Set 1; F(1,17) = 6.581, p<0.05 for Set 2). Overall, KO showed more activity in the dark phase (Fig. 2B and C). Both WT and KO showed gradual decrease in activity in the dark phase during the course of the seven days, whereas activity in the light phase remained low.

In the open field test, both genotypes had similar exploration distance in fifteen minutes (WT vs. KO: 5421.1±833.1 cm vs. KO 5211.4±320.5 cm, p = 0.619 for Set 1; 6283.5±1401.9 cm vs. 5563.6±1189.3 cm, p = 0.232 for Set 2). The mean distance traveled in one minute could not be distinguished by genotype throughout the fifteen minutes of the experiment. The total time spent in the center of the arena was similar between WT and KO in Set 1 (224.7±117.5 s vs. 242.0±70.8 s, p = 0.695, t-test), however KO tended to stay longer in the center position in Set 2 (163.8±41.7 s vs. 281.6±73.5 s, p<0.01, t-test).

In the light-dark box test, the results varied between Set 1 and Set 2 (as summarized in Table S1). Therefore, we decided that the test does not delineate behavioral phenotypes of RAGE KO mice.

Both WT and KO displayed comparable behavioral patterns in the elevated plus maze test under 70 lx condition (Set 1) and 40 lx condition (Set 2). The proportion of the time spent in the open arm (WT vs. KO: 14.5±19.6% vs. 24.5±28.1%, p = 0.597 for Set 1; 23.1±15.4% vs. 13.3±14.0%, p = 0.09 for Set 2; Mann-Whitney's U-test,) and the relative frequency of open arm entry (31.0±16.9% vs. 32.1±24.3%, p = 0.971 for Set 1; 31.6±11.0% vs. 24.0±14.0% p = 0.307 for Set 2, Mann-Whitney's U-test) were not significantly different.

**Figure 3:**
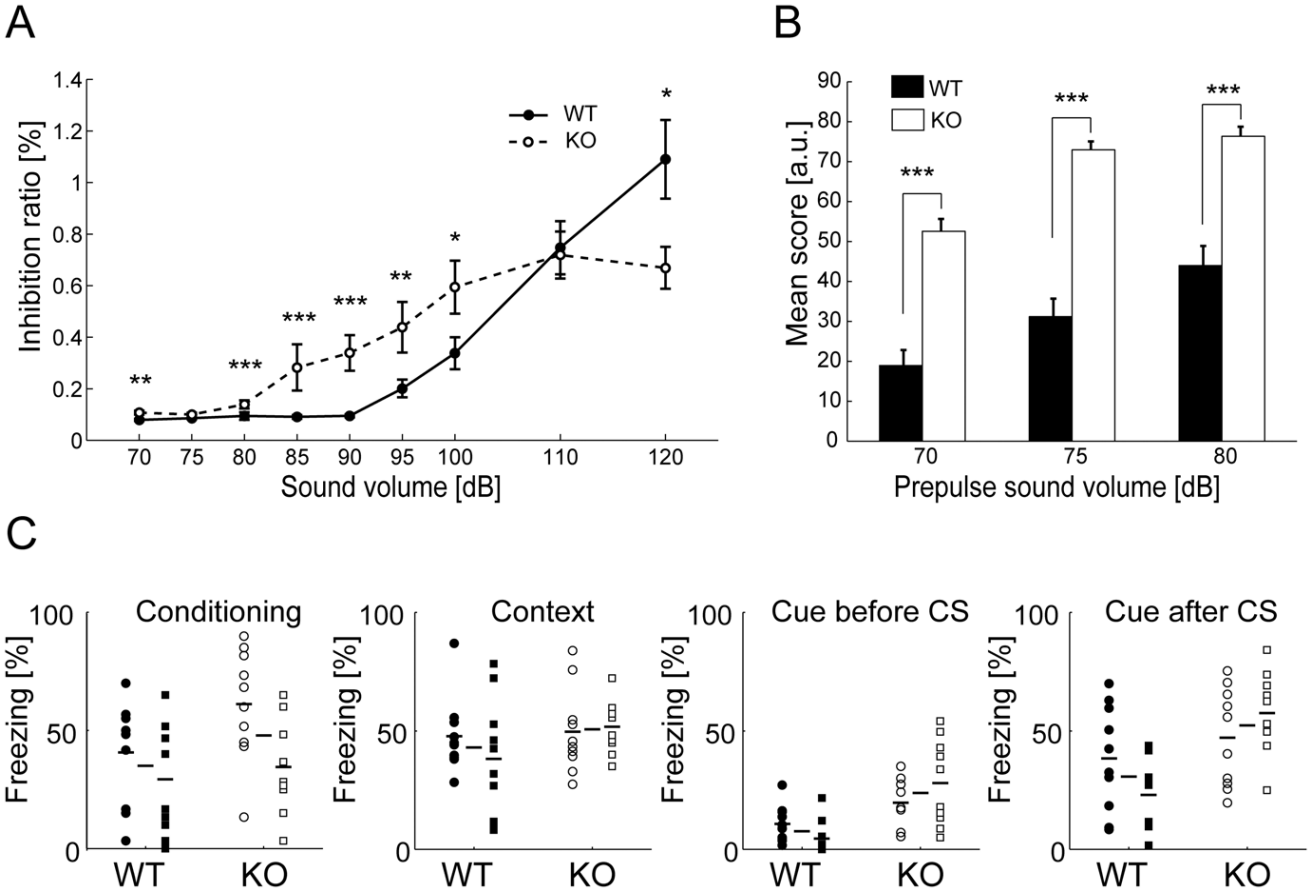
Auditory startle response assessment resulted in a higher sensitivity to auditory signal and cue-dependent fear memory was affected in RAGE KO. (A) KO mice are more sensitive to auditory stimulation (Set 1 & 2 combined). (B) Prepulse inhibition showed the response is more inhibited in KO mice. Abscissa values indicate the volume of prepulse tones. Data are mean±S.E.M. for A and B. * p<0.05, ** p<0.01, *** p<0.001. (C) Four stages of freezing response in the classical fear conditioning test are plotted. The freezing responses at final bin (30 s period, 1 min after the final (second) shock) of the conditioning phase (Conditioning) were not significantly different between WT and KO mice. Both WT and KO show similar freezing responses in the context test (Context). In the cue test, there is a significant difference in the freezing response in the cue test cage *without* the conditional stimuli (Cue before CS). Overall, KO mice show a higher sensitivity to the conditional auditory stimuli in the cue-dependent test (Cue after CS). Set 1 and Set 2 data are represented by circles and squares, respectively. Horizontal bars correspond to the median values for Set 1, Set 1 & 2, and Set 2. See the main text for detailed statistics.

Auditory startle response assessment resulted in a higher sensitivity to auditory signal in KO. In both Set 1 and 2, WT were virtually irresponsive to auditory signals up to 90 dB, whereas KO showed response from 85 dB (Fig. 3A). The WT displayed startle response at 95 dB or larger. Prepulse inhibition showed a clear difference between WT and KO (Fig. 3B). For all the tested prepulse tones (i.e. 70 dB, 75 dB and 80 dB), KO startle response was more inhibited by the prepulse sound (t-test, p<0.05 for all of the cases for Set 1, p<0.01 for all cases for Set 2). Similar results were obtained with a startle stimulus of 110 dB tested in Set 2, in that KO showed significantly more prepulse inhibition for all the tested prepulse tones (p<0.05 for 70 dB, p<0.01 for 75 and 80 dB).

Morris water maze test was done to test animals' spatial learning ability. There was no significant difference in the total distance traveled to find the target during four days of training between the genotypes (two-way ANOVA for genotype, F(1,54), p = 0.962 for Set 1; F(1,54), p = 0.06 for Set 2). Similarly, the probe test did not yield any performance differences in the target ratio measured by stay time (WT vs. KO: 34.3±12.1% vs. 32.4±9.6, p = 0.734 for Set 1; 26.8±19.9% vs. 36.3±13.0%, p = 0.273 for Set 2; Mann-Whitney's U-test) or in the target ratio measured by number of crosses (44.0±15.2% vs. 34.8±16.4%, p = 0.167 for Set 1; 35.3±33.3% vs. 34.9±21.9%, p = 1.00 for Set 2; Mann-Whitney's U-test).

The experimental animals were tested for fear conditioning. During conditioning trials, both WT and KO showed similar freezing response after electric foot shocks (final bin freezing behavior percentage WT vs. KO: 40.7±21.6% vs. 61.2±23.5%, p = 0.070 for Set 1; 29.3±23.1% vs. 34.5±19.2%, p = 0.956 for Set 2, Mann-Whitney's U-test, Fig. 3C). In the assessment of context dependence of the fear, both genotypes appeared to elicit similar degree of freezing behavior (25.5±11.8% vs. 28.7±15.9%, p = 0.705 for Set 1; 38.3±24.8% vs. 51.9±10.8%, p = 0.131 for Set 2, Mann-Whitney's test). There was no difference in the freezing response tested against the conditioned sound cue Set 1, (38.4±22.3% vs. 47.1±20.7, p = 0.406, Mann-Whitney's U-test), however KO displayed significantly higher freezing response in Set 2 (23.0±15.8% vs. 57.5±16.9% p<0.01). Combined population statistics show the difference overall is significant (p<0.01). Interestingly, there is a statistical difference in the freezing response in the cue test cage without the conditional stimuli (10.7±7.7% vs. 19.6±10.9%, p<0.05 for Set 1; 4.5±7.1% vs. 28.0±18.0%, p<0.01 for Set 2, Mann-Whitney's U-test).

## Discussion

Among the series of behavioral tests, the most striking behavioral difference was observed in the home cage activity. RAGE KO mice displayed ∼30% higher activity in darkness on day 1 and persistently higher activity during the seven days of observation. In addition, auditory startle response assessment resulted in a higher sensitivity to auditory signal in KO mice. The higher sensitivity to auditory signal provides an explanation for the increased prepulse inhibition ratio in KO animals and auditory cue-dependent classical fear conditioning. The animals' curiosity or anxiety should be excluded from the subject of the difference, as the open field test and the elevated plus maze test yielded no significantly different scores.

Our results indicate that deletion of RAGE has minimal effects on the animals' spatial learning ability (as tested with Morris water maze and context-dependent classical fear conditioning). Therefore, it appears that RAGE does not have a critical importance in synaptic plasticity of the hippocampus and the associated areas. In fact, long-term potentiation in the entorhinal cortex has been reported to be not affected in RAGE KO mice [16]. By contrast, genetic manipulations of S100B, a ligand for RAGE, result in more visible effects on learning and memory. S100B KO mice improve performance in spatial learning and become more sensitive to context-dependent fear conditioning [12], whereas S100B overexpressing transgenic mice have inferior performance in spatial learning [17]. The behavioral differences between RAGE KO and S100B KO imply that RAGE may not be a crucial receptor of S100B for learning and memory. It is, however, noted that attenuation of kainate-induced gamma oscillations in S100B KO [18] has recently been demonstrated to be dependent on activation of RAGE [13], suggesting a role of RAGE in hyperactive brain states. One potential caveat is that the mice used in the current study have been backcrossed eight times to C57BL6, so that the expected percentage of genetic material from the original strain is below 0.4%.

As the KO phenotypes were dependent on presentation of sensory stimulus, RAGE may play an active role in sensory organs or the brain. Immunohistochemical localization of RAGE in the brain has remained controversial to date [19], [20], [21], [22]. Furthermore, esRAGE, a soluble and secretory form of RAGE, could play an important role. Interestingly, reduced immunoreactivity against esRAGE in CA3 hippocampal neurons were found in Alzheimer's patients [23]. Future investigations on localization of membrane bound and soluble forms of RAGE, as well as RAGE induced biochemical pathways shall further identify the role of RAGE in the central nervous system.

As activation of RAGE accelerates pathological progression of diabetes or Alzheimer's disease, therapeutic treatments to attenuate activation of RAGE have been suggested [24] and experimented in animal disease or inflammation models [8], [25], [26], [27]. Our results raise a possibility that systemic therapeutic treatments to occlude RAGE activation may have adverse effects as demonstrated by the home cage activity and prepulse inhibition behavioral tests. Further investigations using mice of different background strains and identification of biochemical pathways that elucidates the behavioral phenotypes are needed for better understanding of RAGE in basal states.

## Materials and Methods

### Subjects

RAGE (−/−) (KO) mice were generated similar to as described in Myint et al. [14]. Briefly, the RAGE mutant mice were originally created using E14.1 ES cells (129 background). After the chimeric mice were made, they were crossbred with Cre-transgenic mice (CD-1 background) that transiently express Cre recombinase in eggs [28]. The resultant RAGE KO mice were then backcrossed to C57BL/6J (Charles River Japan) for eight generations. Two independent populations of ten mature male RAGE KO mice and ten mature male wild type (WT) RAGE (+/+) mice were used. Littermates and non-littermates were mixed. The first group consisted of mixed yet age-matched population ranging from postnatal eight to eighteen weeks. In the second group, the age was more tightly matched so that the ages of the mice were all eight weeks. Mice were genotyped prior to the behavioral experiments, but the identities of the mice were not exposed to the experimenter during the behavioral experiments. Mice were housed individually before transferring to the behavioral laboratory. The light condition was 12/12 hour light-dark cycle with light phase starting at 8:00 a.m. The temperature and humidity of the laboratory were maintained at 22–23°C and 50–60%, respectively. Food and water were freely available for entire period of the home cage activity measurement and when the mice were housed in their home cage. Large blunt tongs wrapped with silicon rubber were used to handle mice to avoid individual variability in the handling procedure. All of the experiments were conducted in the light phase.

### PCR Genotyping

Tissue samples from the ear were dissolved in a buffer containing (50 mM KCl, 10 mM Tris-HCl, pH 8.3, 2 mM MgCl2, 0.1 mg/ml gelatin, 0.45% NP-40, 0.45% Tween-20, 0.5 mg/ml proteinase K) at 55°C for overnight. The lysate, dNTP mixture, TaKaRa Ex Taq, Taq buffer and the following three primer were mixed; 5′-CCAGAGTGACAACAGAGCAGAC-3′ (primer 1), 5′-GGTCAGAACATCACAGCCCGGA-3′ (primer 2), and 5′-CCTCGCCTGTTAGTTGCCCGAC-3′ (primer 3) (nucleotides 73915-73936, 74523-74544, and 74881-74902 in GenBank accession no. AF030001, respectively). The thermocycle for the PCR reaction consisted of the following sequences: 94°C (1 min) followed by 35 cycles of 95°C (30 s), 62°C (30 s), 72°C (30 s), followed by 74°C (10 min) incubation. The mixtures were separated in 1% agarose gel and the band images were captured by a CCD camera system (Dolphin-View, Wealtec).

### Column Chromatography and Western Blotting

A polyclonal anti-RAGE antibody (H-300, Santa Cruz Biotech. Inc.) was coupled to HiTrap NHS-activated HP Columns (GE Healthcare) according to the manufacturer's instructions. Tissue homogenates (1 ml) from lung (0.18 g or 0.2 g) and brain (0.5 g or 0.5 g) of RAGE KO or WT mice, respectively, in tissue lysis buffer of 50 mM Tris-HCl (pH 7.5), 1% TritonX-100, 150 mM NaCl, and proteinase inhibitors (10 KIU/ml aprotinin, 1 µg/ml leupeptin, 1 µg/ml pepstatin A, 1 mM benzaminidin, and 1 mM EDTA) were applied to the HiTrap-anti-RAGE antibody column previously equilibrated with the lysis buffer. After washing with a 5 bed volume of the equilibration buffer, bound proteins were eluted with 0.1 M glycine–HCl (pH 2.5). The eluate was precipitated with 10% trichloroacetic acid (TCA) at 4°C for 15 min. The pellet was re-suspended in SDS-polyacrylamide gel electrophoresis (SDS-PAGE) sample buffer (62.5 mM Tris–HCl (pH 6.8), 2% SDS, 5% 2-mercaptoethanol, 10% glycerol, and 0.002% bromophenol blue) and boiled at 95°C for 5 min. Proteins in the lysates were resolved by SDS-PAGE (5–20%) and transferred onto a polyvinylidene fluoride membrane (Millipore Corp.). The membranes were incubated with a polyclonal anti-RAGE antibody [14] and an IRDye 680 donkey-anti-rabbit antibody (LI-COR Biosciences, NE) was used as a second antibody. The signal was monitored using a LI-COR Odyssey IR imaging system (Lincoln, NE).

### Behavioral Tests

The experimental animals were subject to a series of behavioral tests performed according to the schedule described in Table 1. The procedure for each behavioral test is described below (further details of the procedures are described in Kato et al. [29]). Dimensions of experimental apparatuses are represented as (width × length × height). After each trial (except the auditory startle response test and the water maze test), the apparatuses were wiped and cleaned with 80% alcohol and damp towel. In the auditory startle response test, holding chambers were washed by tap water, wiped by paper towel, and dried after each trial. All experimental protocols were approved by the RIKEN Institutional Animal Care and Use Committee.

**Table 1:** Behavioral battery test schedule.

Set 1
Day	Time	Behavioral paradigm
1	AM	Introduction to behavioral experiment room
	PM	Home cage activity test started (at 15:00)
8	PM	Home cage activity test finished
14	PM	Open field test (15 min, 70 lx)
15	PM	Light-Dark box test (10 min)
19	PM	Elevated plus maze test (5 min, 70 lx)
21	PM	Startle response & PPI test (120 dB)
22	PM	Startle response & PPI test (120 dB)
25	AM/PM	Water maze test: training day 1
26	AM/PM	Water maze test: training day 2
27	AM/PM	Water maze test: training day 3
28	AM/PM	Water maze test: training day 4
29	PM	Water maze test: probe test
33	PM	Fear conditioning test (conditioning trial)
34	PM	Fear conditioning test (context trial)
35	PM	Fear conditioning test (cued trial)
Set 2		
Day	Time	Behavioral paradigm
1	AM	Introduction to behavioral experiment room
	PM	Home cage activity test started (at 15:00)
8	PM	Home cage activity test finished
14	PM	Open field test (15 min, 70 lx)
20	PM	Open field test (15 min, 250 lx)
26	PM	Light-Dark box test (10 min)
32	PM	Elevated plus maze test (5 min, 40 lx)
39	PM	Startle response & PPI test (110 dB)
40	PM	Startle response & PPI test (110 dB)
46	PM	Startle response & PPI test (120 dB)
47	PM	Startle response & PPI test (120 dB)
53	AM/PM	Water maze test: training day 1
54	AM/PM	Water maze test: training day 2
55	AM/PM	Water maze test: training day 3
56	AM/PM	Water maze test: training day 4
57	PM	Water maze test: probe test
60	PM	Fear conditioning test (conditioning trial)
61	PM	Fear conditioning test (context trial)
62	PM	Fear conditioning test (cued trial)

#### Home cage activity measurement

Spontaneous activity of mice in their home cage was measured using a 24 channel activity monitoring system (O'Hara, Tokyo, Japan). Cages were individually set into the compartments made of stainless steel in the negative breeding rack (JCL, Tokyo, Japan). A piezoelectric sensor was equipped on the ceiling of each compartment to detect the mouse movements. Activity counts represent the number of active time bin (approximately 0.20–0.25 s each) in which spontaneous activity including locomotor activity, rearing and other voluntary stereotypic movements were detected. Home cage activity was measured for seven consecutive days during which bedding materials were not changed.

#### Open field test

Open field test apparatus was placed in a small sound-proof room (185×185×225 cm). The apparatus consisted of four white plastic boxes (50×50×40 cm), two electric fans for ventilation and background noise (35 dB), white LED light source (70 lx at the center of the field) which served as the sole source of illumination during the experiment. For each box, a CCD camera is attached on the ceiling for monitoring mice. Mice were individually introduced at the center of the arena and were allowed to move freely for 15 min. Distance traveled (cm) and % duration of staying at the center area of the field (30% of the field) were adopted as the indices, and they were collected every 1 min.

#### Light-dark (L-D) box test

A light-dark box system was equipped in the same sound-proof room as the open field test. The light box was made of white plastic (20×20×20 cm) and illuminated by LEDs (250 lx at the center of the box) and a CCD camera was equipped on the ceiling, and the dark box was made of black plastic (20×20×20 cm) and an infrared camera was equipped on the ceiling. The light box and dark box was connected by a gate for transition on the center panel between the light box and dark box (5×0.5×3 cm) with a slide door. Mice were individually introduced into the light box, and the door of the tunnel automatically opened after two seconds. Then mice were allowed to move freely for ten min. Total distance traveled, % distance traveled in the light box, % duration staying in the light box, number of the transitions between the light and dark boxes and the latency to first enter the dark box were measured.

#### Elevated plus maze test

An elevated plus maze consisted of a pair of closed arms (25×5×15 cm) and a pair of open arms 25×5×0.3 cm) was placed in the same sound-proof room as the open field test. The floor of each arm was made of white plastic and the walls of the closed arms and ridges of the open arms were made of clear plastic. The closed arms and open arms were arranged orthogonally. The apparatus was elevated 60 cm above the floor and illuminated at 70 lx at the center platform of the maze (5×5 cm). Mice were individually put on the center platform facing to an open arm, and then mice were allowed to move freely in the maze for 5 min. Total distance traveled, % time stayed in the open arms, % number of the open arm entry were measured.

#### Auditory startle response

Each mouse was put into a small cage for startle response (30 or 35 mm diameter, 12 cm long) and set on the sensor block in a sound-proof chamber (60×50×67 cm) with dim illumination (10 lx at the center of the sensor block). White noise (65 dB) was presented as background noise. Experimental session began after the mouse was acclimatized to the environment for five min. In the first session, only startle stimuli (SS, 120 dB, 40 ms) were presented for ten times in random inter-trial intervals (ITI, 10–20 s). In the second session, startle response to stimuli at various intensities were assessed. Five rounds of 70 to 120 dB white noise stimuli (in 5 or 10 dB increments, 40 ms) were presented in quasi-random order and random ITI. In the prepulse inhibition (PPI) session, mice experienced five types of trials; no stimulus, SS only, and prepulse (20 ms, lead time 100 ms)-SS pairings with three different prepulse volumes (70 dB, 75 dB, and 80 dB). Each trial repeated ten times in quasi-random order and random ITI. In the final session, only SS were again presented for ten times in random ITI.

#### Morris water maze test

A standard Morris' water maze test was performed [30]. Briefly, a circular maze made of white plastic (1 m diameter, 30 cm depth) was filled with white-colored water to about 20 cm in depth (22 to 23°C). There were some extra-maze landmark cues (i.e., calendar, figure, plastic box) that were visible from the mice in the maze. Mice underwent six trials per day for four consecutive days. Each acquisition trial was initiated by placing an individual mouse into the water facing the outer edge of the maze at one of the four designated starting points in quasi-random order. The submerged platform remained constant for each mouse throughout testing. A trial was terminated when the mouse reached the platform, and the latency and distance swam were measured. Mice that did not reach the platform within 60 s were placed on the platform for extra 30 s before being returned to their home cage. The inter-trial interval was about 6 min. After the four day training, a probe test was conducted. In the probe test, the platform was taken away and each mouse started from the point opposite from the target platform to swim for 60 s. The distance swam, the number of crossings the position of the target platform and other three platforms, time staying in the quadrants of the four platforms were measured.

#### Classical fear-conditioning

Classical fear conditioning test consisted of three parts; a conditioning trial, a context test trial, and a cued test trial. Fear conditioning was carried out in a clear plastic chamber equipped with a stainless steel grid floor (34×26×30 cm) connected to an electric shock generator. A CCD camera was equipped on the ceiling of the chamber. White noise (65 dB) was supplied as an auditory cue (CS). The conditioning trial consisted of a 2 min exploration period followed by two CS-US pairings separated by 1 min each. A US (foot-shock: 0.5 mA, 2 s) was administered at the end of the 30 s CS period. A context test was performed in the same conditioning chamber for three min in the absence of CS. The cued test was performed in an alternative context with different chamber (triangular shape, white color walls, 0–1 lx brightness, solid floor with thin bedding materials). The cued test consisted of a 2 min exploration period to evaluate the nonspecific contextual fear, followed by 2 min CS period (no US) to evaluate the acquired cued fear. Rate of freezing response (immobility excluding respiration and heartbeat) of mice was measured as an index of fear memory.

### Data Analysis

Behavioral experiments with mouse tracking information were analyzed with custom-modified ImageJ software (O'Hara, Tokyo, Japan). ImageJ is public domain software available from NIH (http://rsb.info.nih.gov/ij). The measured analyzed values are represented in terms of mean±standard deviation throughout the manuscript, unless otherwise noted.

## Supporting Information

Table S1 Behavioral scores for the light-dark box test.
(0.03 MB DOC)
