# Supplementary material for: Deletion of RAGE Causes Hyperactivity and Increased Sensitivity to Auditory Stimuli in Mice
Source: PLoS One. 2009 Dec 15;4(12):e8309. doi: 10.1371/journal.pone.0008309 (PMC2788702; doi:10.1371/journal.pone.0008309)
Supplement: Table S1 — Behavioral scores for the light-dark box test. (0.03 MB DOC) [file pone.0008309.s001.doc]

## Supporting Information

Table S1. Behavioral scores for the light-dark box test

| *Light-Dark Box* | Set 1 | | | Set 2 | | |
| --- | --- | --- | --- | --- | --- | --- |
|  | WT | KO | Sig. | WT | KO | Sig. |
| Exploration distance  (Light) [cm] | 574.9±214.6 | 839.8±216.5 | * | 1097.8±133.8 | 831.1±121.7 | *** |
| Exploration distance  (Dark) [cm] | 1158.9±189.0 | 1150.4±218.2 | n.s. | 1429.6±243.4 | 1354.0±142.7 | n.s. |
| Number of transitions  [times] | 25.6±8.2 | 37.1±11.7 | * | 47.1±8.0 | 42.3±7.8 | n.s. |
| Latency to enter dark  [s] | 48.5±56.0 | 53.7±39.6 | n.s. | 18.6±12.4 | 15.4±13.9 | n.s. |
